# Psychometric properties of the Last-7-Day Sedentary Time Questionnaire (SIT-Q-7d): Testing the validity and reliability among general population

**DOI:** 10.1186/s12889-022-14262-x

**Published:** 2022-10-08

**Authors:** Fatemeh Bakhtari Aghdam, Sepideh Aziz-Zadeh, Saeed Musavi, Mahdieh Abbasalizad-Farhangi

**Affiliations:** 1 Road Traffic Injury Research Center, Tabriz University of Medical Sciences, Tabriz, Iran; 2 Department of Health Education and Promotion, Tabriz University of Medical Sciences, Tabriz, Iran; 3 Department of Statistics and Epidemiology, Faculty of Health, Tabriz University of Medical Sciences, Tabriz, Iran; 4 Department of Community Nutrition, Faculty of Nutrition, Tabriz University of Medical Sciences, Attar Neyshabouri Street, Tabriz, Iran

**Keywords:** Reliability, Validity, SIT-Q-7d questionnaire, Sedentary behavior, Iran

## Abstract

**Backgrounds:**

Sedentary behavior (SB) is an independent risk factor causing chronic diseases. Previous studies compared sitting time mostly with physical activity. The present study aimed to evaluate the validity and reliability of the Last-7-Day Sedentary Time Questionnaire (SIT-Q-7d) in Iran. Moreover, SB was assessed among the study participants.

**Methods:**

The current validity study was conducted among 290 subjects (51.7% males vs. 48.3% females) with a mean age of 34.81 ± 9.63 years in Poldasht, Iran. Sampling was done using simple random sampling and the data were collected using the SIT-Q-7d. To confirm the validity of the questionnaire, forward–backward translation method, content validity, and construct validity were used. Furthermore, temporal stability was calculated by the test–retest method and internal consistency coefficient (ICC).

**Results:**

Our results confirmed the content validity of the questionnaire (content validity score: 0.90 and content validity index: 0.80). Using exploratory factor analysis (EFA), seven factors of SB were identified as follows: eating while sitting down, doing domestic affairs, screen time, leisure time, studying books, watching TV, and attending family gatherings. The reliability of the questionnaire was confirmed using Cronbach’s alpha (α = 0.7). In addition, temporal stability was confirmed by test–retest method and ICC was 0.9 (95% CI: 83–97).

**Conclusion:**

Our results confirmed that the Persian version of SIT-Q-7d is a reliable and valid tool for assessing SB.

**Supplementary Information:**

The online version contains supplementary material available at 10.1186/s12889-022-14262-x.

## Background

Sedentary behavior (SB) is any seated or reclining behavior, whilst awake, with energy expenditure at or below 1.5 metabolic equivalents [1]. Extensive advances in the modern world and industrial development have transformed the human life to a sedentary lifestyle and increased the desire for urban life [2]. As a result, SB has increased in different societies, especially in developed countries [3, 4]; therefore, it is now considered as one of the most serious health challenges in healthcare systems worldwide [5]. SB increases the risk of cardiovascular diseases (CVDs), diabetes, obesity, and mortality [6].

According to the reports from the United States and Australia, more than half of the waking day (over 50%) of adults is spent as sedentary [7]. In Iran, 65% of the adults have a sedentary lifestyle. In addition, the results of measuring physical activity showed that about 70–80% of the population are physically inactive [8, 9].

It is essential to measure SB so as to monitor the public health in the community level and evaluate the efficacy of the interventional programs [10–15]. Currently, there is no valid and reliable tool in Iran for evaluating sedentary lifestyle. So, it is essential to develop a valid and reliable questionnaire for SB measurement in small- or large-scale populations [16]. The measurement tool must be valid and reliable so that the researcher can collect the related data, evaluate the given theories, and answer the research questions through analyzing the data.

Numerous instruments have been used to measure the SB in different countries, including Sedentary Behavior Questionnaire (SBQ), the Last-7-Day Sedentary Time Questionnaire (SIT-Q-7d) [1], Past-Day Adults’ Sedentary Time (PAST) Questionnaire, and Sedentary Time Questionnaire (SIT-Q) [17–19]. In this regard, while some questionnaires focused on the domain-specific SB, few questionnaires evaluated some domains of them; for example Clark BK et al*.* evaluated the leisure time SB [20], while some others evaluated workplace sitting behavior [21, 22]; also sitting time in specific age groups and not general population [23, 24] or specific health conditions like overweight or obese individuals [25], or patients with cancer [26] were evaluated. These factors will limit the generalizability of psychometric properties to be used in general population. Therefore, it is essential to develop a tool to evaluate SB across all age groups and health conditions with special emphasize on all the domains of SB. Among the mentioned tools, it seems that SIT-Q-7d [1] is the most suitable questionnaire, because it uses the short frame of reminiscence (last seven days) and lets the individual to remember his/her ordinary actions. In addition, other questionnaires do not assess all domains, which may negatively affect the estimation of total sitting time. This questionnaire is a short questionnaire that collects the information of specific behaviors and incorporates more intra-individual variability in SB [1]. Also, regarding the scarcity of SB measurement tools in Iran, the present methodological study aimed to evaluate the validity and reliability of SIT-Q-7d questionnaire in Iran.

## Methods

### Participants

In the current methodological study, we included 290 participants aged over 18 years in Poldasht, Iran from 25 January to 9 July, 2020. This city has two regions with two health centers. Using a simple random sampling method, the individuals meeting the inclusion criteria were selected from a list of people covered by the health centers. Since the information about Iranian families is kept in the health centers, we used the existing lists in these centers for sampling. The exclusion criteria included not answering all the questions in the questionnaire, having psychological problems, and having physical and mobility problems.

### Last-7-Day Sedentary Time Questionnaire (SIT-Q-7d)

The SIT-Q-7d was developed by Wijndaele et al*.* in Australia in 2014, and its validity and reliability were approved [1]. This tool has five domains, which measure the amount of time that people spent sitting or lying down in the last seven days. The first domain examines the average daily hours people spend on sleeping and napping (e.g.: On average, how long did you nap per day?). The second domain evaluates the amount of time people spend sitting for breakfast, lunch, and dinner (e.g.: On average, how long did you sit for breakfast per day?). The third domain measures the time people spend sitting during transportation, such as travelling in a car, bus, train, on a motorbike, etc. (e.g.: On average, how long did you sit while travelling to and from your job per day?). The fourth domain evaluates the time people spend sitting during work, study, and volunteering (e.g.: On average, how long did you spend sitting or lying down for studying per day?). The fifth domain measures the screen time and sitting hours spent on other activities, such as looking at screens and monitors (e.g.: On average, how long did you spend sitting for playing computer game).

In each domain, the sedentary time during weekdays and weekend days is calculated by specific time periods (less than 15 min, 15–30 min, 30–45 min, 45 min – 1 h, 1–1.5 h, 1.5–2 h, 2–2.5 h, 2.5–3 h, 3–4 h, 4–5 h, 5–6 h, 6–7 h, and more than 7 h). For the second domain, the sedentary time during the weekdays and weekend days is evaluated by specific time periods (less than 15 min, 15–30 min, 30–45 min, 45 min – 1 h and more than 1 h a day). In any domain, the SB is calculated using the total minutes of SB and calculating their means. To calculate total SB, the total minutes of SB in each domain for the weekdays and weekend days are added. The validity of SIT-Q-7d was confirmed in four stages, including forward–backward translation, face validity, content validity, and construct validity.

### Forward–backward translation

Backward translation was applied to remove the confounding effects of cultural context in which the questionnaire is applied [27]. The original questionnaire was independently translated from English into Persian by two health professionals fluent in both Persian and English languages. Then, a consolidated version of the questionnaire was produced. Any inconsistency between the two translated versions was resolved by discussion or through the help of a third translator. Finally, two independent English translators reviewed and translated the questionnaire back to English to ensure both versions are similar.

### Face and content validity

The Persian version of the questionnaire was distributed among ten experts in the fields of health education and promotion, epidemiology, physical education, and sport sciences, and evaluated for face validity and content validity (appropriateness of the questions to the research aims). The necessary changes were made in terms of appearance, full clarity of questions, and categorization of SB areas according to the Iranian context. The first, second, third, and fourth sections of the questionnaire (‘sleeping and napping’, ‘meals’, ‘transportation’, and ‘work, study, and volunteering’) were used without any changes; but the fifth section (‘screen time and other activities’) was conducted separately to measure the SB precisely. Our panel of experts stated that the sitting time spent on doing household tasks, watching screens and TV, studying books, listening to music or radio, and socializing, which had been included in the fifth section of the original English questionnaire, had to be studied as a separate questionnaire in the Persian version. Thus, these domains were separated from each other and distributed among participants as a separate questionnaire. After modifying the Persian version and applying the comments of the panel of experts, content validity of the quantitative section was evaluated through asking multiple-choice questions from the experts to assess the clarity, simplicity, relevance, and necessity of each question in the Persian questionnaire. Finally, content validity index (CVI) was obtained based on the first three indicators (clarity, simplicity, relevance) and content validity ratio (CVR) was calculated based on the indicator of ‘necessity’. In the present study, CVI was 0.80 and CVR was 0.90, which confirmed the content validity of the tool according to the recommendations by the World Health Organization (WHO) [28].

### Sample size calculation

Sample size adequacy was analyzed through considering three approaches. First, exploratory factor analysis (EFA) was used to analyze the data and evaluate construct validity. Since in this approach the correlation between the items forms the bases of analysis, the ratio of sample size to the number of parameters in the model must be at least five to one, or preferably ten to one. Hence, because there were 29 items in the questionnaire, the sample size had to be more than five times the number of the questions in the questionnaire. Thus, we considered 290 subjects as the sample size [29]. Also, as Everitt BS et al*.* [30] recommended that 5–15 respondents for each question would give optimum sample size, we chose ten respondents for each question and 290 subjects were adequate. Also, according to the guidelines by MacCallum C et al*.* [31] for minimum sample size requirements, because the communalities for all of the variables was around 0.50, sample sizes between 100 and 200 would be sufficient [32].

### Evaluation of construct validity and statistical analysis approach

In the present study, EFA was used to evaluate construct validity. Sampling adequacy for factor analysis was performed by Kaiser–Meyer–Olkin (KMO) measure and Bartlett’s test of sphericity. Any factor with an eigenvalue equal to one or above was considered significant for factor extraction. If the loading criterion was 0.4 or more, a principal component analysis (PCA) using varimax rotation was used for factor extraction.

Using the Stata Statistical Software (Version 17; Stata Corp), confirmatory factor analysis (CFA) was used to evaluate how well the EFA model fits into the observed data. To apply fit indices, the comparative fit index (CFI), the Tucker-Lewis index (TLI), the root mean square error of approximation (RMSEA), and the standardized root mean square residual (SRMSR) were applied with cut-off points of adequacy as follows: CFI > 0.80; TLI > 0.80; RMSEA and SRMSR with acceptable values of zero to one [33–35]. To analyze the collected data, the Statistical Package for the Social Sciences (SPSS Inc., Chicago, USA; version 25) was used. For quantitative data, we used the mean, standard deviation, and median (Q1-Q3**)**, and for qualitative data, we used the frequency and the percentage. Kolmogorov–Smirnov test was used to check data distribution. Accordingly, none of the SB variables had normal distribution. Therefore, the comparison of paired samples was performed using the Wilcoxon signed-rank test.

### Data collection

To evaluate the socioeconomic condition, the 6-item socioeconomic status (SES) tool was used. Sadeghi et al*.* confirmed the validity and reliability of this tool. The items of this tool include ‘occupation of the head of household as the main source of income’, ‘education of the head of household’, ‘household’s monthly income’, ‘local value of residence’, ‘value of personal car’, and ‘proportion of medical expenses of the household to all costs’. In this tool, a score below 11.97 indicates a low SES, a score between 11.98 and 16.96 indicates an average SES, and a score over 16.97 indicates a high SES [36]. Demographic variables, including age, gender, weight, height, body mass index (BMI), marital status, education (under diploma, diploma, college degree), and occupation (housewife, employee, retired, freelancer, etc.) were also evaluated.

## Results

### Construct validity

**Fig. 1:**
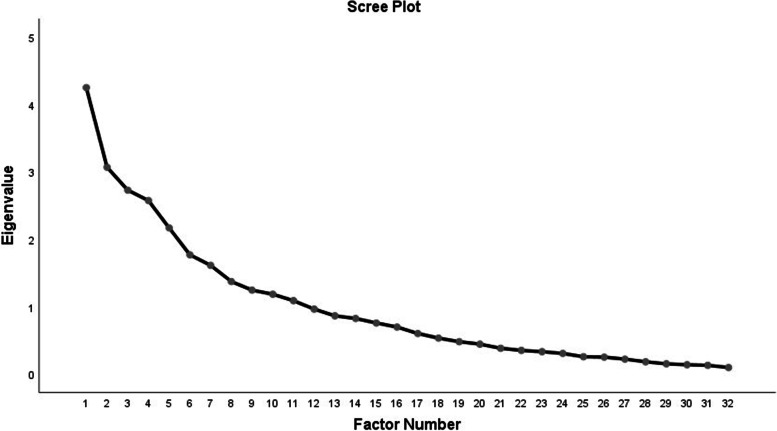
Scree plot for determining factors of the last-7-day sedentary time questionnaire (SIT-Q-7d)

Since the missing rate in all the items of the questionnaire were less than 5% and the missing mechanism was completely random, we removed the missing data in the final analysis. The results showed that, based on KMO test, the amount of this statistic was 0.63, indicating a sufficient sample size [37, 38]. Based on this statistic, the values < 0.5 indicated weak EFA, 0.5–0.7 moderate EFA, 0.7–0.8 good EFA, 0.8–0.9 great EFA, and > 0.9 excellent EFA [39]. In this questionnaire, Bartlett’s test was also significant (*P* < 0.001) and showed explorable relationships between variables. In other words, the variables or sub-factors extracted by EFA were correlated with each other [40]. The PCA revealed a seven-factor solution for the 29 items based on an eigenvalue greater than one. The seven-factor solution explained the 56.81% variance. The scree plot also showed a seven-factor solution (Fig. 1).

Table 1 shows the loads related to rotated factors. Factor load is the correlation coefficient between the factor and the question, and its value indicates the strength of association (priority of the question for the factor). As can be seen, grouping the factors to factor 1 (sitting time for meals), factor 2 (sitting time for doing household tasks), factor 3 (screen time), factor 4 (sitting time for leisure activities), factor 5 (sitting time to watch TV, read books, etc.), factor 6 (sitting time for socializing), and factor 7 (sitting time for other activities) was correct and the results from the data were correlated with the given theory. The original English questionnaire also included the sitting time for ‘occupation’ and ‘transportation’, but since half of the samples in the current study were unemployed, we considered this time as zero for them. Meanwhile, the employed people spent some time in sitting position due to daily commutes, which was calculated as zero for the unemployed people. Since 109 samples did not have a private car and the research environment was a small city, we considered their transportation as active. Thus, the existing data may not be powerful enough to evaluate the occupation and transportation domains. However, we included these two domains in the questionnaire based on the experts’ ideas. To assess the fitness of the model obtained from the EFA, the CFA was conducted on 29 questions of the final questionnaire. The fit of the model is shown in Sup. Figure 1. Fit indices were calculated using covariance matrixes. All fit indices proved the moderate goodness of tests. The relative chi-square (χ2/df) was equal to 3.91 (*P* < 0.001) and the RMSEA was equal to 0.190 (90% CI = 0.194–0.270). All comparative indices of the model, including CFI and TLI, exceeded the value of 0.80 (0.891 and 0.876 respectively).

**Table 1:** Results of Factor Loads for the SIT-Q-7d

Items	Factor 1	Factor 2	Factor 3	Factor 4	Factor 5	Factor 6	Factor 7
Sitting during meals							
Sitting for lunch at week days	0.80						
Sitting for lunch at weekend	0.68						
Sitting for breakfast at weekend	0.66						
Sitting for dinner at weekend	0.65						
Sitting for dinner at week days	0.64						
Sitting for breakfast at week days	0.63						
Sitting at domestic							
Caring for children and elderly at weekend		0.82					
Caring for children and elderly at week days		0.76					
Sitting for doing household at week days		0.72					
Sitting for doing household at weekend		0.71					
Sitting for screen time							
Using computer at week days			0.70				
Playing sedentary computer game at week days			0.70				
Playing sedentary computer game at weekend			0.69				
Using computer at weekend			0.63				
Sitting for L–T							
Listing to music or radio at week days				0.93			
Listing to music or radio at weekend				0.81			
Watching TV and reading							
Reading or performing							
Performing at weekend					0.69		
Reading at week days					0.67		
Reading at weekend					0.61		
Performing at week days					0.50		
Socializing							
Socializing at week days						0.83	
Socializing at weekend						0.73	
Sitting for other activities							
Sitting for other activities at week days							0.82
Sitting for other activities at weekend							0.63
Watching TV at week days							0.46

*SIT-Q-7d* Last-7-day sedentary time questionnaire, *L–T*, Leisure time

^*^ Factor loading higher than 0.4 is acceptable

### Reliability

To assess the temporal stability, the questionnaire was answered by 20 individuals in two weeks. Considering the minimum acceptable internal consistency coefficient (ICC) of 0.50 and the expected level of ICC equal to 0.90 with two raters (α = 0.05 and β = 0.2), the sample size was calculated to be 18 individuals. Finally, considering the 10% drop-out rate, 20 individuals were selected to assess the reliability [41–43]. For reliability assessment, internal consistency of the questionnaire was calculated by Cronbach’s alpha, and the temporal stability was calculated by ICC coefficient. The reliability of the questionnaire was confirmed by Cronbach’s alpha (α = 0.7). The values for subscales ‘sitting time for meals’, ‘sitting time for doing household tasks’, ‘screen time’, ‘sitting time for leisure activities’, ‘sitting time to watch TV, read books, etc.’, ‘sitting time for socializing’, and ‘sitting time for other activities’ are provided in Table 2. The temporal stability of this tool was also confirmed by test–retest method and ICC was satisfactory 0.9 (95% CI: 0.83–0.97). The ICCs for each of the above subscales have been shown in Table 2.

**Table 2:** The Cronbach’s alpha and ICC for each of the domains of SIT-Q-7d

SIT-Q-7d domains	Cronbach’s alpha	ICC
Sitting during meals	0.802	0.84, 95% CI = 0.80–0.89
Sitting at domestic	0.616	0.89, 95% CI = 0.86–0.91
Sitting for screen time	**0.672**	0.80, 95% CI = 0.78–0.85
Sitting for L–T	**0.780**	0.78, 95% CI = 0.75–0.82
Watching TV and reading	**0.801**	0.91, 95% CI = 0.86–0.93
Socializing	**0.681**	0.85, 95% CI = 0.82–0.92
Sitting for other activities	0.681	0.75, 95% CI = 0.70–0.81
Total score	0.7	0.9 (95% CI: 0.83–0.97)

*SIT-Q-7d*, Last-7-day sedentary time questionnaire, *ICC* Internal consistency coefficient, *CI*, Confidence interval

### Demographic findings of participants

Out of 290 participants with a mean age of 34.8 years, 150 (51.7%) were male and 140 (48.3%) were female. Moreover, 130 (44.9%) participants had a college degree, 106 (36.5%) had a high school diploma, and 54 (18.6%) were under diploma. Also, 226 (77.9%) were married and 190 (65.5%) were employed. The mean sitting time during the weekdays and weekend days without considering the domain of ‘sleeping and napping’ was 6.7 (2.3) and 5.3 (2.2) hours, respectively. The highest mean was related to ‘reading books’ and ‘watching TV’ with the mean sitting time of 2.9 (2.4) hours in a day followed by ‘screen time’ with the mean sitting time of 2.7 (3.17) hours in a day. Meanwhile, the lowest mean was related to ‘occupation’ with the mean sitting time of 1.2 (1.63) hours in a day. In the domain of ‘household tasks’ and ‘transportation’, the sitting time was significantly higher during the weekdays compared to weekend days (Table 3).

**Table 3:** The Mean and medians of different domains of sedentary behavior

**Sedentary Behavior**		**Mean (SD)**	**Median (Q1-Q3)**	*P-value
**Sleeping and napping (hour)**	**Weekdays**	8.63 (1.74)	8.62 (2.13–11.19)	0.528
	**Weekend days**	8.73 (1.73)	8.75 (2–12)	
**Meals (hour)**	**Weekdays**	1.25 (0.63)	1.13 (0.92–3.17)	0.123
	**Weekend days**	1.24 (0.65)	1.09 (0.8–2)	
**Doing household chores(hour)**	**Weekdays**	2.27 (2.94)	1.12 (0.86–3.25)	0.006
	**Weekend days**	2 (2.94)	0.75 (0.35–2.88)	
**Screen time (hour)**	**Weekdays**	2.78 (3.17)	1.5 (1.1- 4.37)	0.325
	**Weekend days**	2.88 (3.01)	1.87 (1.76–4.5)	
**Leisure time (hour)**	**Weekdays**	1.46 (1.96)	0.75 (0.47–1.5)	0.125
	**Weekend days**	1.53 (1.94)	0.75 (0.39–2.5)	
**Reading books & watching TV (hour)**	**Weekdays**	2.93 (2.4)	2.25 (1.83–3.75)	0.090
	**Weekend days**	2.92 (2.41)	2 (1.75–3.8)	
**Socializing (hour)**	**Weekdays**	1.60 (2.12)	0.75 (0.7–2.5)	0.060
	**Weekend days**	1.69 (2.15)	0.76 (0.69–2.9)	
**Other activities (hour)**	**Weekdays**	1.21 (1.75)	0.37 (0.1–2.5)	0.146
	**Weekend days**	0.65 (1.20)	0.37 (0.18.75)	
**Transportation (hour)**	**Weekdays**	2.48 (2.26)	1.6 (1- 4.41)	0.006
	**Weekend days**	1.2 (1.02)	1.7 (0–4.2)	
**Occupation (hour)**	**Weekdays**	1.2 (1.63)	1.06 (.75–1.76)	-
	**Weekend days**	-	-	
**Total sitting time (hour)**	**Weekdays**	6.7 (2.3)	5.2 (1.2–6.9)	0.225
	**Weekend days**	5.3 (2.2)	4.3 (1.5–7.2)	

^*^P-value was performed by Wilcoxon signed-rank test

The results of present study showed that women had significantly higher SB than men in domains of ‘doing household tasks’ and ‘socializing’, but SB was higher among men in the domain of ‘transportation’. Moreover, married people had a higher SB in domains of ‘doing household tasks’ and ‘occupation’ compared to unmarried people; however, unmarried people had a significantly higher SB in the domain of ‘screen time’. Furthermore, people with higher education had a higher SB in the domain of ‘screen time’, but a significantly lower SB in ‘socializing’. In the domains of ‘meals’ and ‘screen time’, people with a higher BMI had a significantly higher SB compared to those with a lower BMI. Although people from lower socioeconomic groups had a lower SB in the domains of ‘watching TV’ and ‘reading books’, they had a higher SB in the domain of ‘transportation’ (Sup. Table 1).

## Discussion

The present study aimed to assess the validity and reliability of the SIT-Q-7d questionnaire. The findings showed that the questionnaire had an acceptable reliability based on test–retest method. Based on Cronbach’s alpha, the reliability of all questions was 0.7 in the most suitable state, which confirms the association of the SB variables in the questionnaire. The reliability of this tool was also confirmed (α = 0.6) by Wijndaele et al*.* [1] in Australia. Regarding temporal stability, the results of test–retest method (95% CI: 0.83–0.97) in two weeks showed that the questions of the questionnaire had a good reliability and could indicate the stability of the results over time.

We used the content analysis and factor analysis to evaluate the reliability of the structure. In content analysis, our panel of experts stated that the sitting time spent on doing household tasks, watching screens and TV, reading books, listening to music or radio, and socializing, which had been included in section five of the original English questionnaire, had to be studied as a separate questionnaire in the Persian version. Thus, these domains were separated from each other in the Persian tool. In factor analysis, seven factors with total variance of 56.81 were identified. The number of factors in current study was higher than the original tool possibly due to cultural differences between Australian and Iranian people. For example, some SB domains such as socializing are more common in Iran than Australia. Furthermore, the number of factors in current study (seven factors) was higher than that of the original tool, which had five factors including ‘sleeping and napping’, ‘meals’, ‘transportation’, ‘work and education’, and ‘screen time and other activities’.

In our study, the first factor load was ‘sitting for meals’, which included the time spent on breakfast, lunch, and dinner during weekdays and weekend days; this is theoretically acceptable and is matched with the English version of SIT-Q-7d questionnaire [17]. The second factor load was ‘doing household tasks’, which included the time spent on caring for children and the elderly and doing the household tasks; this is theoretically acceptable and is matched with the fourth factor of the English tool. Furthermore, in the International Physical Activity Questionnaire (IPAQ), there is a section entitled ‘domestic activities’, which is similar to our Persian version of the SIT-Q-7d questionnaire [44]. The third factor load was ‘screen time’, which included using computer and playing video games; this is theoretically acceptable and is matched with all the three questionnaires of SIT-Q-7d (17), PAST (19), and SBQ [18]. The fourth factor load was ‘leisure time’, which included listening to music and radio. It should be noted that in Iranian culture socializing is not considered as a leisure activity and it is a kind of sub-culture; hence, visiting parents and relatives is a social norm recommended in Islam. In a study conducted by Razavi in Iran, about 65.6% of participants socialized and interacted with each other in a face-to-face manner [45]. The fifth factor load was ‘watching TV and reading books’ during the weekdays weekend days, which is theoretically acceptable and is matched with both SBQ and PAST questionnaires [18, 19]. The sixth factor load was ‘socializing’, which was calculated separately following the ideas of the panel of experts; as mentioned above, this factor is acceptable in cultural norms of Iran. The seventh factor load was ‘other activities’, which included watching TV while doing other tasks such as speaking on the phone, doing an art work, or practicing a skill.

The PAST questionnaire, whose validity was confirmed by Clark et al. in Australia [1], has the following seven factors: occupation, transportation, watching TV, using computer, reading books, leisure time, and other activities. In the PAST questionnaire, except for two factors of occupation and transportation, the other factors are matched with those of the present study.

The SIT-Q questionnaire [17] has seven factors, including meals, transportation, work and volunteering, caring for children and the elderly, watching TV, using computer, and leisure time. In this questionnaire, except for occupation and transportation, the other factors are matched with those of the present study.

The SBQ questionnaire has nine factors, including watching TV, playing video games, listening to music and radio, talking on the phone, using computer for emails, chatting, etc., reading books and newspapers, playing musical instruments such as piano, doing an artistic work or practicing a skill, and commuting by bus and car while sitting [18]; five factors of this questionnaire are matched with those of current study.

According to our results, the Persian version of SIT-Q-7d used in this study has a good validity and can be utilized in studies for the evaluation of SB. In addition, the average time of nocturnal sleeping and daily napping was eight hours, which was consistent with the results of the studies carried out by Catherine et al*.* in Australia (1), Mary Carskadon et al*.* in the USA [46], and a review study by Gulia et al*.* [47]. Sleeping and resting are vital physiologic needs and if they are not met, man's life might be endangered [48]. On average, humans spend roughly one-third of their lives asleep. An adult person needs at least eight hours of sleep daily [49]. We used the CFA model to examine whether the hypothesized model fits the data. The CFA results supported the seven-factor model of the EFA model and had moderate fitness.

## Conclusion

The results of the present study indicated that the highest SB among the population of Poldasht, Iran belonged to the domain of ‘sleeping and napping’ followed by ‘watching TV’. Moreover, our results confirmed the content validity and construct validity of the Persian version of the SIT-Q-7d questionnaire. In addition, both internal consistency and temporal stability of the questionnaire were acceptable; therefore, the Persian version of the SIT-Q-7d questionnaire can be utilized in future studies for the evaluation of SB lifestyle.

The total sitting time with the inclusion of sleeping time in this research was about 10 h daily, which was consistent with the results of the study by Catherine et al*.* [1]. The results of the study by Kai et al*.* [5] in Japan showed that Japanese people had a lower SB (five hours in a day), which was lower than the time in our study. This difference is possibly due to the different tools used in the two studies. Kai et al*.* [5] used Sedentary Lifestyle Questionnaire (SLQ), which evaluated the sedentary time only in such domains as occupation, transportation, watching TV, using computer, and reading books; but our study evaluated the sitting time spent on sleeping, meals, household tasks, leisure time, and socializing as well. In other words, the number of SB domains examined in the study by Kai et al*.* was less than that in the current study. Another reason might be the cultural differences between Japanese and Iranian people in sedentary lifestyle. The results of present study showed that females had a significantly higher SB than males in the domains of ‘household tasks’ and ‘socializing’, which is in line with the results of the study by Bossink and Vlaskamp [50]. This might be attributed to the fact that women are more involved in doing household tasks than men. Another probable reason is that Poldasht is a small city and lacks recreational places for women, which leads to the increase of family and friendly gatherings among them. As a result, the sitting time among females is increased.

Furthermore, our results indicated that SB was significantly higher among males in the domain of ‘transportation’, which is similar to the results reported by Dori et al*.* [18]. This might be due to the fact that men usually work out of their houses and have to commute by public transportation, which increases their SB.

The results of the present study showed a significant difference between the married and unmarried individuals in the domains of ‘household tasks’ and ‘occupation’, so that the sitting time of married people was higher than unmarried subjects. This might be due to the household responsibilities of married people, which obliges them to involve in household tasks and work more significantly compared to unmarried people; this is consistent with the findings reported by Van der Ploeg et al*.* [51]. Regarding ‘screen time’, the SB was significantly higher among unmarried people compared to married ones, which is in line with the study by Thanamee et al*.* [37]. This might be due to different responsibilities between the married and unmarried people, because married people are usually busier than unmarried ones and they have to limit their use of mobiles and electronic devices. In addition, unmarried people, due to their younger ages, use mobiles and other electronic devices more frequently, which is the main cause of their higher SB in this domain.

The results of this study also revealed that SB in the domain of ‘screen time’ was significantly higher among people with higher educational levels. This may be attributed to the fact that people with lower educational levels usually have non-desk jobs, which requires physical activity, and they have less free time for using mobiles and electronic devices. Another reason might be that people with lower educational levels have less digital knowledge, which reduces their SB in this domain.

In this research, there was a significant difference between the level of income and SB in such domains as leisure time, reading books, and watching TV, so that people with lower income had a higher SB in the mentioned domains. This might be attributed to the fact that people with lower income cannot afford to go shopping or visit sightseeing places very often; thus, they spend most of their time watching TV and reading books. This is consistent with the results of the study by Ussery et al*.* [38], that demonstrated a direct relationship between a higher level of income and a more physically active lifestyle.

According to our results, in the domain of transportation, there was a significant difference between SB and having a private car, in a way that people without a private car and those with a low-price car had a significantly lower SB compared to those owing a high-price car. This might be due to the fact that Poldasht is a small city and its inhabitants prefer riding a bike or walking to using public transportation.

In the current study, a significant difference was seen regarding the number of children and SB in such domains as doing household tasks and watching TV, so that SB increased by increasing the number of children. This is also in line with the results of the study by Thanamee et al*.* [37]. Although we did not study the effect of children’s age on SB in the current study, it might be said that parents with younger children have a lower SB, because they have to care for them.

There was also a significant difference between the domains of ‘meals’ and ‘screen time’ with the BMI, so that people with a higher BMI had a higher SB. Similar to our results, LaCroix et al*.* [52] showed a reverse and significant relationship between physical activity level and BMI.

This study had some limitations. First, we did not perform concurrent validity. So, we recommend future studies to consider this issue in their methodologies. Second, in some of the study domains, we observed a relatively low value for Cronbach's alpha, so that four values were below the required level of 0.7. This might be due to the relatively low internal consistency and low number of questions in some of these domains (e.g., three items for other activities, two items for socializing, four items for domestic issues, and four items for screen time), but the overall Cronbach's alpha was more than 0.7, which was acceptable.

## Supplementary Information

**Additional file 1: Sup. Table 1. **The Association between demographic variables and different domains of sedentary behavior (*n*=290). **Sup. Figure 1.** The results of structural equation modelling for the confirmatory factor analysis of last-7-day sedentary time questionnaire (SIT-Q-7d); LWD, Sitting for lunch at week days; LWD, sitting for lunch at weekend; BWN, Sitting for breakfast at weekend; DWN, Sitting for dinner at weekend; D W. D, Sitting for dinner at week days; BWD, Sitting for breakfast at week days; WN, weekend; WD, week days.

## Data Availability

The data of the current paper cannot be shared publicly due to the regulations proposed by Tabriz University of Medical Sciences. However, the data are available for the researchers throughout the world by email with reasonable request from the corresponding author.
