# Supplementary material for: Psychometric properties of the Last-7-Day Sedentary Time Questionnaire (SIT-Q-7d): Testing the validity and reliability among general population
Source: BMC Public Health. 2022 Oct 8;22:1880. doi: 10.1186/s12889-022-14262-x (PMC9547410; doi:10.1186/s12889-022-14262-x)
Supplement: Supplementary file 1 — Additional file 1: Sup. Table 1. The Association between demographic variables and different domains of sedentary behavior (n=290). Sup. Figure 1. The results of structural equation modelling for the confirmatory factor analysis of last-7-day sedentary time questionnaire (SIT-Q-7d); LWD, Sitting for lunch at week days; LWD, sitting for lunch at weekend; BWN, Sitting for breakfast at weekend; DWN, Sitting for dinner at weekend; D W. D, Sitting for dinner at week days; BWD, Sitting for breakfast at week days; WN, weekend; WD, week days. [file 12889_2022_14262_MOESM1_ESM.docx]

**Supplementary Material**

**Title: Psychometric properties of the last-7-day sedentary time questionnaire (SIT-Q-7d): Testing the validity and reliability among general population**

**Sup. Table 1.** The Association between demographic variables and different domains of sedentary behavior (n=290)

| **Variables** |  | **N (%)** | **Sleeping & napping** | **Meals** | **Household chore** | **Screen time** | **Leisure time** | **Reading books & watching TV** | **Socializing** | **Other activities** | **Transportation** | **Occupation** |
| --- | --- | --- | --- | --- | --- | --- | --- | --- | --- | --- | --- | --- |
| **Gender** | Male | 150 (51.7) | 8.8 (7.6-9.6) | 2.1  (1.3-3) | 0.37 (0-1.8) | 4.6  (1.3-8.5) | 1.5  (.3-1.9) | 4.7 (3-7.7) | 1.5 (.3-3) | .75  (0-2.2) | 2.2 (1-3.5) | 3.7 (0.75-5.6) |
|  | Female | 140 (48.3) | 8.6 (7.3-9.6) | 2.3  (1.6-3.3) | 5.3 (2.2-11.3) | 3.5  (.9-7.5) | 1.5  (1.3-6) | 5.06 (3-9.1) | 2.5 (.75-6) | .75  (0-3.3) | 1.7 (0-4.2) | 1.3 (0-3.2) |
|  | *P*-value |  | 0.144 | 0.313 | 0.001 | 0.608 | 0.322 | 0.057 | 0.001 | 0.522 | 0.020 | 0.124 |
| **Marital status** | Single | 64 (22.1) | 8.6 (7.9-9.3) | 2.3  (1.5-3.6) | 0.37 (0-2.06) | 7.5  (2.1-10.9) | 1.5  (.37-4.8) | 4.2 (2.4-7.6) | 1.5 (.28-3.4) | 1.5  (.28-3.4) | 1.2 (0-1.5) | 1.5 (0-4.2) |
|  | Married | 226 (77.9) | 8.6 (7.1-9.6) | 2.1  (1.5-3.1) | 2.7 (.5-7) | 3.6  (.5-8.1) | 1.5  (.37-5) | 5 (3-8.5) | 1.5 (0.37-5) | .75  (0-2.8) | 2.1 (0.6-6.3) | 2.1 (0-6.2) |
|  | *P*-value |  | 0.393 | 0.455 | 0.001 | 0.002 | 0.454 | 0.062 | 0.234 | 0.407 | 0.132 | 0.002 |
| **Education** | Under Diploma | 54 (18.6) | 8.6 (7.3-9.8) | 2.05  (1.2-2.5) | 1.7 (.8-4.7) | 1.6  (0-3.9) | 0.93  (0-4) | 5 (3-8.5) | 6.2 (0.75-6.2) | 0.75  (0-3.2) | 1 (0-3) | 0.9 (0-2.5) |
|  | Diploma | 106 (36.5) | 8.6 (7.4-9.8) | 2.1  (1.5-3.4) | 1.5 (0-6.3) | 4.7  (.75-9) | 1.5  (.5-2.6) | 6 (2-8.2) | 2.5 (0.75-4.1) | .75  (0-2.6) | 1.2 (.75-3.5) | 2.5 (.5-6.9) |
|  | College Degree | 130 (44.9) | 8.9 (7.5-9.7) | 2.4  (1.5-2.6) | 1.5 (0-5.1) | 7.5  (2.7-9.1) | 1.5  (.5-3.5) | 5.1 (2.3-9.1) | 1.5 (0.13-5) | .75  (.37-2.4) | 0.9 (0-1.5) | 1.02 (0-3) |
|  | *P*-value |  | 0.794 | 0.082 | 0.233 | 0.002 | 0.781 | 0.913 | 0.019 | 0.618 | 0.123 | 0.152 |
| **BMI** | Low | 7 (2.4) | 8.2 (7.2-9.9) | 2.1  (1.2-3.2) | 3.2 (.75-5.2) | 4.5  (1.5-6.2) | 3.4  (1.7-5.2) | 3.5 (1.3-6.2) | 2.9 (1-3.5) | 1.8  (.75-3) | 2.8 (0-4) | 2.9 (0-4.5) |
|  | Normal | 66 (22.7) | 8.5 (7.1-9) | 2.6  (1.2-2.5) | 3.5 (0-6) | 6.6  (0-9) | 3.1  (1.2-5) | 5.2 (2.1-8.3) | 2.1 (0.75-4) | 1.9  (0.75-3.5) | 2.6 (1.2-3.9) | 2.5(1.2-6) |
|  | High | 123 (42.4) | 8.4 (7.2-9.2) | 3.2  (1.6-4.5) | 5.3 (1.2-7.1) | 7.1  (1.5-10.2) | 2.2  (1.6-4) | 3.9 (0-6.5) | 1.9 (.88-2.5) | .75  (0-2.5) | 3.4 (1.5-4.6) | 3.2 (0.75-6) |
|  | Obese | 94 (32.5) | 8.9 (7.3-10) | 3.3  (1.8-4.8) | 4.1 (3.3-6.5) | 7.5  (3.1-11.2) | 2.9  (.75-4.5) | 3.8 (0.8-6.5) | 3.2 (1.5-4.2) | 1.9  (0-3.2) | 2.5 (1.4-5.6) | 2.6 (1.5-5.2) |
|  | *P*-value | 0.325 | 0.020 | 0.129 | 0.129 | 0.002 | 0.302 | 0.192 | 0.231 | 0.919 | 0.291 | 0.206 |
| **Age** | 18-20 | 24 (8.4) | 8.4  (7.3-9.2) | 2.1  (1.2-2) | 3.4 (2.9-5) | 6  (4.1-7.2) | 2.6  (1.9-3.2) | 2.6 (1.8-4) | 1.6 (.88-2.5) | 2.6  (1.2-3) | 1.7 (1.5-4) | 1.5 (0-3.2) |
|  | 20-40 | 156 (53.7) | 8.2  (7.1-9.6) | 3.1  (1.6-4.2) | 5.3 (4.2-5.9) | 5.3  (3.1-6.5) | 1.7  (0.9-2.8) | 3.2 (2.6-4) | 3 (1.2-5) | 3.9  (1.7-5) | 2.3 (0-4.5) | 3.2 (1.2-4) |
|  | 40-59 | 110 (37.9) | 8.2  (8-9.2) | 3.3  (2.1-3.9) | 3.5 (2.9-4.9) | 4  (0.5-5.3) | 3  (1.7-4.2) | 5 (3.1-5.9) | 4.2 (2.1-6.2) | 1.8  (0.75-4) | 1.5 (0.75-2.5) | 1.8 (1-2.5) |
|  | *P*-value |  | 0.581 | 0.213 | 0.001 | 0.919 | 0.231 | 0.008 | 0.547 | 0.193 | 0.512 | 0.415 |
| **SES** | Low | 118 (40.8) | 8.3  (7.2-9.4) | 1.7  (0.9-2.5) | 4.2 (2.6-6.5) | 3.5  (1.5-5.6) | 1.5  (0.3-4.6) | 3.5  (1.2-4.6) | 2.3 (1.5-4) | 0.75  (0-2.2) | 2.5 (1.3-5.2) | 2.5 (1.7-5.2) |
|  | Moderate | 86 (29.6) | 8 (0-9) | 2.5  (1.3-3.6) | 2.3 (0-4) | 4.6  (2.2-6.3) | 3.6  (1.5-5.2) | 3.6  (0-4) | 1.7 (0.3-3.2) | 1.2  (0.4-3.2) | 2.1 (0-4.2) | 1.2 (0.3-3.5) |
|  | High | 86 (29.6) | 8.5 (7.1-9.6) | 3.3  (1.9-5.2) | 2.1 (1.5-3.2) | 6.5  (3.2-9.5) | 4.5  (3-6.5) | 4.6  (3.1-6.9) | 4 (0.6-5.2) | .9  (.3-1.7) | 1.2 (.3-2.7) | 1.9 (0-2.6) |
|  | *P*-value |  | 0.512 | 0.421 | 0.912 | 0.125 | 0.231 | 0.002 | 0.921 | 0.305 | 0.020 | 0.202 |

**BMI, body mass index; SES, socio-economic status.**

**Sup. Figure 1.** The results of structural equation modelling for the confirmatory factor analysis of last-7-day sedentary time questionnaire (SIT-Q-7d); LWD, Sitting for lunch at week days; LWD, sitting for lunch at weekend; BWN, Sitting for breakfast at weekend; DWN, Sitting for dinner at weekend; D W. D, Sitting for dinner at week days; BWD, Sitting for breakfast at week days; WN, weekend; WD, week days.
